# Religious versus Conventional Psychotherapy for Major Depression in Patients with Chronic Medical Illness: Rationale, Methods, and Preliminary Results

**DOI:** 10.1155/2012/460419

**Published:** 2012-06-13

**Authors:** Harold G. Koenig

**Affiliations:** ^1^Departments of Psychiatry and Medicine, Duke University Medical Center, Box 3400 Medical Center, Durham, NC 27705, USA; ^2^Department of Medicine, King Abdulaziz University, Jeddah 21413, Saudi Arabia

## Abstract

This paper (1) reviews the physical and religious barriers to CBT that disabled medically ill-depressed patients face, (2) discusses research on the relationship between religion and depression-induced physiological changes, (3) describes an ongoing randomized clinical trial of religious versus secular CBT in chronically ill patients with mild-to-moderate major depression designed to (a) overcome physical and religious barriers to CBT and (b) compare the efficacy of religious versus secular CBT in relieving depression and improving immune and endocrine functions, and (4) presents preliminary results that illustrate the technical difficulties that have been encountered in implementing this trial. CBT is being delivered remotely via instant messaging, telephone, or Skype, and Christian, Jewish, Muslim, Buddhist, and Hindu versions of religious CBT are being developed. The preliminary results described here are particular to the technologies employed in this study and are not results from the CBT clinical trial whose findings will be published in the future after the study ends and data are analyzed. The ultimate goal is to determine if a psychotherapy delivered remotely that integrates patients' religious resources improves depression more quickly than a therapy that ignores them, and whether religious CBT is more effective than conventional CBT in reversing depression-induced physiological changes.

## 1. Introduction

Depression is a major public health problem. Based on a joint study conducted by the Harvard School of Public Health and the World Health Organization, depression was the leading cause of disability in the world (measured by years of life lived with disability) in 1990 [1] and, in 2020, is expected to be the world's second leading cause of disability, surpassed only by cardiovascular disease [2]. The lifetime prevalence of depression in the USA is 20% in women and 10% in men [3]. 

Depression is widespread in patients with chronic disabling medical illness. While the point prevalence of major depression among the general population in the United States is 7% [4], this figure increases to 10% to 45% among patients with medical illness depending on setting [5–8]. Not surprising, the use of antidepressants by primary care physicians has increased dramatically in recent years [9]. Treating with antidepressants, while lifesaving for many, comes with it the risk of side effects in medically frail patients and increases the risk of drug interactions with medications prescribed for nonpsychiatric illness [10]. This is especially true since major depression is already a potent risk factor for disease morbidity, with depressed medical patients having double the mortality of those without depression [11, 12]. Furthermore, medications are expensive, and managing side effects or drug interactions increases the cost further.

Psychotherapy has also been shown to benefit depressed medical patients. The kinds of depression seen in primary care medicine are often situation-specific and due to life changes brought on by physical illness, including day-to-day problems with functioning at home and work. Thus, psychotherapeutic approaches focused on helping patients adjust to difficult real life circumstances are useful in depressed patients seen in primary care settings. Not surprising, psychological approaches such as cognitive-behavioral therapy (CBT) have been particularly effective in treating depression in medical patients, often done by addressing the maladaptive beliefs and thoughts related to medical illness that initiate and maintain depression [13–16].

The aims of this paper are to (1) review the physical and religious barriers to psychotherapy that disabled medically ill-depressed patients face, (2) discuss research on the relationship between religious involvement and depression, and their relationships to physiological changes influencing the course of medical illness, (3) describe an ongoing randomized clinical trial designed to overcome physical and religious barriers to psychotherapy in the medically ill by utilizing remote delivery methods for CBT that integrate religious resources into psychotherapy, and (4) present preliminary results that illustrate the technical difficulties that we have encountered in implementing this trial. 

## 2. Barriers to Psychotherapy

While psychological treatments for depression are known to be effective, there are numerous barriers to referral, compliance, and follow-up of persons with depressive disorders. These include physical, cultural, and religious barriers. Physical barriers to psychotherapy for medically ill-disabled patients are formidable. Medical patients with mobility problems or those who are home bound may have trouble accessing psychotherapy (i.e., traveling to therapists' offices, sitting in waiting rooms, etc.). To address this barrier, online and telephone approaches to delivering CBT have been developed, and are effective in and acceptable to medical patients with depression [17, 18]. 

### 2.1. Online Communications 

Online communications are now widespread in the United States and around the world. Of 6.8 billion people, 25% (1.7 billion) use the Internet [19]. Of the 341 million people in North America, nearly 75% (252 million) use the Internet. In 2005, close to 75 million Americans used e-mail and search engines on an average day (including 29% of those over age 50) [20, 21]. That number has increased considerably since then, not to mention the millions who now have a MySpace or Facebook account (74% of all Americans ages 18 to 34, and 25% of those ages 55 or older), or the estimated 15 million who use Twitter [22]. One of the fastest growing groups of Internet users is older adults; 77% of all persons over age 55 use the Internet to search for healthcare information [23]. 

### 2.2. Online CBT 

Individual cognitive-behavior therapy (CBT) can be offered online by a therapist using instant messaging, during which client and therapist communicate in real time with typewritten responses. Benefits of this approach include flexibility and optimal use of patient and therapist time, outreach to patients for whom travel to treatment centers is difficult for reasons of geography or disability, and access to foreign language therapists. Furthermore, this approach is acceptable to depressed patients, and therapy without face-to-face contact may encourage greater disclosure [24]. 

The Lancet published the first report of a randomized controlled trial (RCT) of therapist-delivered online CBT for moderately severe depression in primary care patients in 2009 [25]. The study found that CBT by this method was significantly more beneficial than usual care provided by primary care physicians. Those benefits were maintained over at least 8 months. This means that a relatively small number of therapists can cover a wide geographical area and be available to patients at a range of times. The way that patients express their feelings may also impact treatment effectiveness. Online CBT takes advantage of the fact that writing about traumatic experiences improves outcomes [26, 27]. This appears especially true when writing about events from a religious viewpoint [28, 29].

### 2.3. Telephone CBT 

 An issue with online CBT, particularly when delivered alone on the Internet without therapist guidance, has been “*dropouts*” during treatment [30]. Even with therapist-delivered online CBT, this remains a problem (dropout rate was 28% in the *Lancet* study). To minimize dropouts and boost effects, it has been recommended that a telephone component be added [31] since telephone therapy is known to increase compliance [32]. Furthermore, structured CBT delivered by telephone has been shown to be both clinically effective and cost effective in treating medical patients with depression [33]. 

## 3. Religion and Depression

Besides physical barriers that depressed disabled medically ill patients encounter when seeking psychotherapy, there are also religious barriers. When religious patients become depressed, their beliefs may interfere with their acceptance of and compliance with conventional treatments, especially psychotherapy. Many such patients shy away from secular psychotherapy because they perceive it as unsympathetic to their religious beliefs [34]. Religious patients may also feel that seeking therapy means abandoning their faith in favor of secular treatments. Finally, religious persons may feel guilty or ashamed about being depressed and thus fail to address it with their clergy and avoid seeking support within their faith community. 

Clergy have traditionally served as front-line mental health providers in communities across the USA, providing nearly as many hours of counseling as does the entire membership of the American Psychological Association [35]. Consider that clergy spend on average 15% of their time in counseling activities, providing over 140 million hours of mental health services each year, not including the activities of nearly 100,000 full-time nuns or chaplains. Furthermore, they do not charge for their services, and there is no stigma associated with this type of counseling. Thus, depressed religious persons often receive their first treatment by clergy or other counselors within their faith community. 

Treatment within the faith community, however, is not always effective, especially for more severe depression, requiring referral to mental health professionals for additional treatment. The problem is that the relationship between clergy and mental health professionals has not always been good. In fact, there is a long history of conflict between religious and mental health professionals, beginning with Freud's description of religion as “the universal obsessional neurosis.” [36] There is often open resistance to consideration of religious beliefs in conventional mental health care, resistance that is clear from a recent discussion among British psychiatrists (see e-letters in response to two recent articles in *The Psychiatrist*) [37, 38]. Negative attitudes toward religion by mental health professionals are not limited to Great Britain. A systematic review of the religious content of DSM-III-R found that nearly one-quarter of all cases of mental illness included religious descriptions [39]. More recent publications by mental health professionals continue to emphasize a lack of concern for patients' religious beliefs, [40, 41] and a recent national survey of USA psychiatrists found that 56% never, rarely, or only sometimes inquire about religious/spiritual issues in patients with depression or anxiety [42]. 

Based on this generally neglectful (and at times disparaging) view held by some mental health professionals toward religion, religious professionals are often reluctant to refer members of their congregation to mental health professionals, especially for psychotherapy that seeks to alter beliefs and attitudes. Failure of clergy to refer may prevent many patients from receiving the treatment they need. Furthermore, if patients are members of a faith community and that community does not reinforce (or perhaps even counteracts) the gains made in psychotherapy, then those gains may not last. 

## 4. Depression, Religion, and Physiological Changes

Whether religion is a resource (as clergy claim) or a liability (as mental health specialists like Freud claim) to depressed medical patients needs to be established before bringing down the barrier that stands between mental health care and religion. Like it or not, religious involvement is important to the vast majority people in the USA and around the world [43]. According to a January 2009 Gallup Poll, 65% of Americans indicated that religion is an important part of their daily life, a figure that increases to over 75% in the southeastern USA [44]. Likewise, according to the Pew Foundation's national survey of 35,000 Americans, 56% indicated that religion was “very important” in their lives, a figure that increases to 69% in the southeastern USA This is especially true for medical patients, who often turn to religious beliefs to cope with illness [45]. 

Literally hundreds of qualitative and quantitative studies document high rates of religious coping behaviors in those trying to cope with medical illness [46, 47]. In some areas of the USA, nearly 90% of hospitalized patients with medical problems use religion to cope, and of those who do, nearly half (45%) report that religion is the *most important* factor that keeps them going [48]. Furthermore, greater religiosity predicts a faster resolution of depressive symptoms in medical patients over time, increasing the speed of remission by 50 to 70 percent overall, but especially in those with persistent physical disability, in whom it predicts a more than 100 percent increase in speed of remission [49–52]. 

Religious involvement has also been associated with positive emotions such as optimism and purpose in life [53], as well as gratefulness, generosity, and altruism [54–56]. These characteristics may enhance well-being and counteract maladaptive cognitions and behaviors that maintain depression [57]. Religious people, however, are not exempt from depression, especially when serious health problems strike. In a study at Duke Hospital, 64% of medical inpatients over age 50 with major depression (diagnosed using the Structured Clinical Interview for Depression (SCID)) indicated they were both spiritual and religious and 76% prayed at least once daily [58]. 

## 5. Consideration of Religious Beliefs in Therapy

Depression not only destroys quality of life but may also affect the physical body by interfering with immune and endocrine functioning. Religious beliefs and behaviors that facilitate coping with life stress may help to normalize those changes.

### 5.1. Physiological Effects of Depression

There is evidence that the alterations in immune and endocrine function associated with depression increase medical morbidity by increasing the risk of infection [59], inflammatory disorders [60], and possibly malignancy [61–63]. This relationship, however, is a complex one that is likely bidirectional in nature [64]. Furthermore, depression is known to stimulate some components of the immune system and suppress others. Stimulation of proinflammatory cytokines can lead to sickness behaviors that resemble depression, which has led to consideration of how immune and endocrine functions influence the pathophysiology of depression, especially when depression develops in a setting of chronic stress [65]. 

Regardless of direction of effect, major depression is associated with a host of immune [66], endocrine [67], and proinflammatory functions [68] that could adversely affect physical health and response to medical treatments. Depression is associated with an altered balance in the Th1/Th2 ratio, that is, higher pro-inflammatory Th1 cytokines (IL-1, IL-12, INF-*γ*), higher pro-inflammatory monocytic cytokines (IL-6, TNF-*α*) [69–71], and lower anti-inflammatory Th2 cytokines (IL-4, IL-10) [72]. Depressed patients also have reduced natural killer (NK) cell cytotoxicity [73–75] and diminished lymphocyte responses to phytohemagglutinin and concanavalin A [76, 77]. 

Impaired immune functions associated with depression also appear to normalize in response to treatment with electroconvulsive therapy (serum TNF*α*) [78], antidepressant drug therapy (serum TNF*α* and CRP) [79], and psychological interventions [80] (due in part to a return of the pro-/anti-inflammatory cytokine balance). A number of randomized clinical trials involving psychological interventions that relieve depression or boost positive emotions have reported improvement in immune and/or endocrine functions. For example, Antoni et al. conducted a 10-week CBT stress-management program in human-immunodeficiency-virus- (HIV-) infected men [81]. The intervention significantly increased naïve CD4+ T cells in blood over a 12-month follow-up among those in the CBT group (*n* = 16) compared to controls (*n* = 9). This effect was mediated by a reduction in depressive symptoms and a decrease in urinary cortisol levels. A more recent study by Antoni et al. examined the effects of a 10-week cognitive behavioral stress management program in 128 women with breast cancer, finding that the intervention significantly lowered serum cortisol, increased Th_1_ pro-inflammatory cytokines (IL-2 and INF-*γ*), and increased IL-2 : IL-4 ratio [82]. 

In another clinical trial, Van Middendorp et al. conducted an emotional disclosure intervention (4 weekly sessions) in 68 nondepressed patients with rheumatoid arthritis, finding a reduction in urinary cortisol, a reduction of serum INF-*γ*, and a trend towards a reduction in IL-6 (*P* = 0.07) [83]. Also, a recent intervention by Lee Berk et al. to increase positive emotions (via mirthful laughter) in 20 high-risk diabetics with hypertension and hyperlipidemia found that the intervention lowered serum epinephrine, norepinephrine, INF-*γ*, CRP, TNF-*α*, and IL-6 [84]. Finally, Roberts et al. found that the hypocortisolism in 41 subjects with chronic fatigue syndrome (a depression-like syndrome) was improved following 15 sessions with CBT [85]; thus, whether cortisol is pathologically high or low, psychological therapies may help normalize levels.

In summary, psychotherapeutic treatments that increase positive emotions (particularly CBT) appear to increase naïve CD4+ cells, increase NK cell cytotoxicity, reduce IL-6, reduce TNF-*α*, reduce CRP, reduce INF-*γ* (sometimes increasing in nondepressed female patients), decrease catecholamines, and normalize (decreasing or increasing) cortisol. 

### 5.2. Physiological Effects of Religion

As noted previously, religious involvement has been associated with better mental health. However, it is also associated with better physical health and greater longevity [86, 87]. The mechanisms that explain the association with physical health are unclear but likely involve behavioral and psychosocial factors operating at least partly through immune/endocrine pathways related to stress [88–90]. There is some evidence that religious involvement is associated with better immune and endocrine functions, although no studies have yet examined the effects of a religious psychotherapy on these functions.

For example, Sephton et al. examined the relationship between religious involvement and immune function in 112 women with metastatic breast cancer [91]. Religious expression was positively related to the total number of circulating T cells (*r* = 0.24, *P* = 0.01) and helper T cells (*r* = 0.23, *P* = 0.01), and controlling for social network size, disease, and medical treatment variables had little effect on these relationships. Investigators also found positive associations between religious expression and cytotoxic T cells (*r* = 0.18, *P* < 0.05) and a trend towards greater NK cell numbers (*r* = 0.14, *P* = 0.07). 

Studying a vulnerable population, Ironson et al. examined the effects of changes in spirituality/religiousness (S/R) following the diagnosis of HIV on CD4 counts (T cells) and viral load during 4 years of follow-up [92]. Hierarchical linear modeling was used to examine the effects of changes in S/R over time with the outcome being slopes of change in CD4 cells and viral load during follow-up. Patients who reported an increase in S/R after diagnosis experienced significantly less decrease in CD4 counts and less increase in viral load during the 4-year follow-up. Results were independent of church attendance and initial disease status, medication use, age, gender, race, education, health behaviors, depression, hopelessness, optimism coping, and social support. In fact, among all significant predictors of CD4 cell count and viral load, change in S/R was the most powerful predictor. A number of other studies have found similar connections between religious involvement and other immune functions (T cells, in particular) [93–95], pro-inflammatory indicators (IL-6) [96–98], and endocrine measures (specifically cortisol) [99–103]. 

In contrast to the numerous observational studies cited perviously, there have been far fewer intervention studies. One that examined nondepressed HIV+ patients reported that a stress management intervention designed to increase spiritual growth led to an increase in lymphocyte proliferation and a 3-fold increase of INF-*γ* [104]. Likewise, Eastern spiritual meditation has been shown to increase antibody response to influenza vaccine [105], alter the ratio of pro-/anti-inflammatory cytokines [106, 107], increase NK cell activity [107], reduce cortisol [107–110], and decrease catecholamine [111, 112] levels (and in one study, there was a trend toward superiority over conventional CBT [113]). 

Thus, psychotherapy for depression that utilizes patients' religious resources in therapy may help to normalize endocrine and immune dysfunctions, perhaps even more so than conventional treatments [114]. Whether or not this is true, however, is completely unknown. The study described below is the first randomized clinical trial to address this gap in the literature and to examine whether religious CBT is equally effective, more effective, or less effective than conventional CBT in depressed patients with chronic medical illness. The effectiveness of religious CBT is suggested both (1) by an earlier study showing that religious CBT was more effective than conventional CBT in religious patients [115] and (2) by a study showing that patients receiving conventional CBT responded more quickly to the therapy if they indicated that religion was important to them [116]. 

## 6. Ongoing Randomized Clinical Trial

Psychotherapy that takes into account patients' religious beliefs as potential resources in therapy may not only help to overcome religious barriers to therapy but may also be more effective in resolving depressive symptoms and reversing depression-induced physiological changes. The efficacy of religious psychotherapy, that is, taking into account the religious beliefs and practices of patients and utilizing them in therapy, however, has yet to be examined in medical settings. Religious CBT has been shown to increase the speed of remission in depressed healthy religious patients and do so beyond that achieved by conventional CBT [117–119]. Likewise, a number of studies that utilized patients' religious beliefs in therapy have reported results superior to secular treatments or usual care, especially in religious patients [120–124]. 

Finally, there is plenty of evidence that many patients in the United States prefer to have their religious beliefs integrated into therapy. For example, one recent study found that 77% to 83% of adults aged 55 or older with depression and comorbid chronic medical illness wished to include religion in their therapy [125]. Likewise, in a survey of a more general population of 74 individuals receiving counseling, Rose et al. found that only 18% of patients said they did not want to discuss religion or spirituality in psychotherapy and only 8% said they wanted to discuss spiritual but not religious issues [126]. Likewise, Kelly found in a national survey that only 19% of respondents did not want to integrate religious/spiritual beliefs and values into therapy [127]. Thus, from the patient's perspective, there appears to be little resistance to utilizing their religious beliefs in therapy.

Benefits resulting from addressing religious beliefs in therapy may stem partly from redirecting attention away from a focus on loss and preoccupation with self and instead centering one's thoughts on positive processes such as gratitude, altruism, and generosity. Such positive cognitions have been shown to predict lower depressive symptoms [128–131]. Furthermore, religious cognitions that promote purpose and meaning in life and a hopeful and optimistic attitude toward circumstances may help to counter the negative cognitions and behaviors associated with depression. Indeed, dozens of studies have reported a link between positive emotions such as optimism and purpose in life, religious involvement, and fewer depressive symptoms [132]. Having a common worldview and explanatory model that gives meaning and purpose to negative life events may also strengthen the therapeutic alliance between patient and therapist, which could contribute to treatment efficacy [133]. This may be particularly true for minority populations who often suffer from disparities in psychiatric care and yet tend to be very religious.

## 7. The Interventions

We are now in the first phase of a randomized clinical trail being conducted at two sites, Duke University Health Systems in the Triangle region of North Carolina and Glendale Adventist Medical Center in Los Angeles County. During Phase I (Rounsaville 1a) [134], we are developing and further refining a manual to guide the delivery of religious CBT and are conducting an open trial to assess subject recruitment and to allow therapists to gain experience with the treatment and method of delivery. In this preliminary phase, religious and conventional CBT are being delivered online by instant messaging, over the telephone, or by Skype to determine which method of delivery is most acceptable, preferred, and likely to be complied with by patients during the randomized clinical trial in Phase II. 

In Phase II (Rounsaville 1b), we will conduct a randomized proof of concept comparison of conventional CBT versus religious CBT that will (1) further demonstrate feasibility of recruitment and subject compliance, (2) confirm the expected clinically meaningful difference (effect size) in the therapies, and (3) show differential effects in reversing depression-induced immune and endocrine functions. In this head-to-head trial, 70 persons who are at least somewhat religious/spiritual and ages 18–85 with an episode of major depression (diagnosed by the Mini International Neuropsychiatric Inventory using DSM-IV criteria), moderate severity of depression based on Beck Depression Inventory (BDI) scores, and at least one chronic medical illness will be randomized to either conventional CBT or religious CBT. Excluded will be patients with significant cognitive impairment, those currently receiving psychotherapy, those that meet criteria on the MINI for psychotic disorder, alcohol or substance abuse, or PTSD, those with a history of bipolar disorder, active suicidal thoughts (passive suicidal thoughts will not exclude), diagnosis of HIV/AIDS, autoimmune diseases, or endocrine disorders likely to affect stress hormone levels, or taking immunosuppressant drugs (due to proposed immune and endocrine analyses). 

The trial will consist of ten 50 min sessions, administered by licensed master's level therapists and delivered over 12 weeks. The primary endpoint will be depressive symptoms on the BDI (clinician-rated scales like the Hamilton or MADRS are not being used because nonclinicians blind to study group will be administering outcome assessments). Subjects will be assessed on the BDI at baseline, 4 weeks, 8 weeks, 12 weeks, and 24 weeks. Immune (pro-inflammatory and anti-inflammatory cytokines) and endocrine (urinary cortisol, epinephrine, and norepinephrine) measures will be assessed at baseline, immediately at the end of therapy (12 weeks), and at 24 weeks from baseline. 

## 8. Preliminary Results

Both interventions are taking a CBT approach documented in treatment manuals. Depressed persons with chronic medical illness have often allowed their health problems and disability (i.e., circumstances) to shape the way they think and view their world. Depression is often maintained by a negative worldview that sees the person's situation as hopeless and without meaning or purpose. Both conventional and religious CBT seek to alter these dysfunctional, maladaptive beliefs, replacing them with positive, realistic beliefs and behaviors that generate positive emotions. 

### 8.1. Conventional CBT

CCBT helps depressed patients understand the links between thoughts, emotions, and behavior. It uses guided discovery, Socratic questioning, and challenge of automatic negative thoughts to help patients identify and appraise their cognitions and determine problematic behaviors. Interventions include activity scheduling, along with practice assignments, in which clients test out ideas and behaviors discussed during sessions. In this arm, therapists will be asked to avoid reference to the participant's faith or religious belief. If religious issues come up, therapists will gently redirect the patient to more secular ways of approaching the issue and, if necessary, will address religious issues in the broadest conventional way possible, relating them to other cognitions/behaviors usually addressed in conventional CBT. We felt justified in doing this because most CBT today does not utilize and integrate the religious beliefs of patients into psychotherapy. 

### 8.2. Religious CBT

Religious CBT follows the same process described for CCBT, except that religious beliefs and motivations will be utilized to stimulate changes in thought and behavior. For example, participants may have obsessive guilt about sin or punishment, report religious doubt, or want to discuss the cognitive and emotional elements of their faith. In RCBT all those issues will be considered fully acceptable as part of therapy, including an emphasis on religious resources. In other words, RCBT uses religious rationales (based on the subject's faith tradition) and religious arguments to counter irrational thoughts and utilizes religious behaviors (involvement in the religious community) to increase supportive relationships. RCBT teaches patients to use their own religious teachings, doctrines, and behaviors to help change maladaptive beliefs, values, and behaviors so as to transform their worldview into one that is meaningful, hopeful, and optimistic, one that is incompatible with depression. RCBT seeks to bolster powerful religious beliefs that promote behaviors such as forgiveness, gratitude, generosity, and altruism (focusing on others and on God) that generate meaning and purpose, optimism, and hope, which neutralize depression. 

The RCBT manual is initially being developed within a Christian framework and will then be adapted to the subject's faith tradition (i.e., versions of the manual for Jewish, Hindu, Buddhist, and Muslim patients). These versions of the manual will be prepared by experienced psychotherapists well versed each of these faith traditions, who will work under the direction of Ken Pargament, a psychotherapist widely renowned for his writings on integrating spirituality into psychotherapy [135]. The therapists who develop the different religious versions will help supervise study therapists while they are treating patients in these faith traditions.

## 9. Conclusions

Preliminary results from Phase I indicate that of the first 82 patients screened for inclusion (from North Carolina and Los Angeles County), 78% had a computer at home, 77% had access to the Internet at home, 75% could type easily and quickly on the computer, 68% had a landline telephone, 95% had a cell phone, but only 30% had Skype (or equivalent program) and a webcam. Most (>80%) preferred therapy by telephone, 9% preferred online therapy, and 9% preferred therapy via Skype. Patients were also asked what method of therapy they were not willing to try; 70% said they were unwilling to try online therapy and 78% were unwilling to try Skype. 

Of the 82 subjects, 18 met inclusion criteria and were randomized to treatment arm (one to online, two to Skype, and 15 to telephone, based on what subjects were willing to do). Technical issues encountered during therapy have been the biggest problem. The telephone route has had the fewest issues; 84% of telephone sessions (delivered by two-person conference call) had no technical difficulties; 8% had a problem with conference call ending or dropping; 5% had difficulty with sound; and one patient's cell phone battery died. Overall, only 0.6% of telephone therapy session time was wasted dealing with technical issues (during the course of 57 therapy sessions). For the single subject who was willing to try the online method thus far, difficulty connecting to the online platform (PsychologyOnline) was a problem for all sessions; overall, 35% of session therapy time was wasted on dealing with this issue. With regard to Skype, only 30% of 20 sessions proceeded without technical difficulties; 30% had difficulty with sound, 25% had difficulty connecting with Skype over the computer; and 5% had trouble with delay in picture, video call dropped, or session could not be recorded. Overall, 7% of therapy session time for Skype was spent dealing with technical issues. These preliminary findings suggest that the most preferred method of delivering the therapy and the one with the least technical difficulties is therapy by telephone, although we need more experience with the online and Skype methods.

## 10. Conclusions

Major depression is a common psychiatric illness, especially prevalent among persons with chronic medical illness, that can adversely affect physical health and medical outcomes by altering immune and endocrine functions. Religion is widespread and often used to cope with medical illness and problems with physical functioning. In this study, we are testing whether a psychological therapy that takes advantage of patients' religious resources improves depression more or less quickly than conventional therapy and reverses the adverse physiological changes associated with depression. 

The results from this study will be important because they are relevant to therapists other than those who explicitly practice pastoral counseling, extending to secular therapists as well. If 65% of Americans indicate that religion is an important part of daily life and 80% of depressed patients wish to include it in therapy, then all therapists (whether they have explicit training in pastoral counseling or not) are likely to encounter patients among their clientele who will prefer this approach. Interestingly, Propst et al. in their study of religious versus conventional CBT found that delivery of religious CBT by secular therapists was at least as effective (if not more so) than religious CBT delivered by religious therapists [136]. 

In their 2009 review of religion in psychotherapy, Post and Wade make two points relevant to this issue [137]. First, although therapists in general are less overtly religious than patients, many therapists have spiritual beliefs that should assist them in appreciating the role that patients' religious beliefs play as a resource in their mental health. Second, while it is helpful for therapists to be well versed in the basic tenets of their patients' religious beliefs, it is not necessary for them to be experts in religion: “instead, approaching religious/spiritual clients with an openness and willingness to engage the religious/spiritual conversation will help clients to feel comfortable expressing their needs.” These authors concluded that religious interventions in psychotherapy can be effectively delivered by therapists with a wide range of religious/spiritual beliefs, not only pastoral counselors. 
